# Communities of Arbuscular Mycorrhizal Fungi in *Pyrus pyrifolia* var. *culta* (Japanese pear) and an Understory Herbaceous Plant *Plantago asiatica*

**DOI:** 10.1264/jsme2.ME12180

**Published:** 2013-04-24

**Authors:** Yuko Yoshimura, Akifumi Ido, Teruyuki Matsumoto, Masahide Yamato

**Affiliations:** 1 The United Graduate School of Agricultural Sciences, Tottori University, 4–101 Koyama-Minami, Tottori 680–8553, Japan; 2 Tottori Prefectural Agriculture and Forest Research Institute, Horticultural Experiment Center, Hokuei, Tohaku, Tottori 689–2221, Japan; 3 Fungus/Mushroom Resource and Research Center, Faculty of Agriculture, Tottori University, 4–101 Koyama-Minami, Tottori 680–8553, Japan

**Keywords:** AML1-AML2, canonical correspondence analysis (CCA), correspondence analysis (CA), Orchard, SSU rDNA

## Abstract

We investigated communities of arbuscular mycorrhizal fungi (AMF) in the fine roots of *Pyrus pyrifolia* var. *culta*, and *Plantago asiatica* to consider the relationship between orchard trees and herbaceous plants in AMF symbioses. The AMF communities were analyzed on the basis of the partial fungal DNA sequences of the nuclear small subunit ribosomal RNA gene (SSU rDNA), which were amplified using the AMF-specific primers AML1 and AML2. Phylogenetic analysis showed that the obtained AMF sequences were divided into 23 phylotypes. Among them, 12 phylotypes included AMF from both host plants, and most of the obtained sequences (689/811) were affiliated to them. Canonical correspondence analysis showed that the host plant species did not have a significant effect on the distribution of AMF phylotypes, whereas the effects of sampling site, soil total C, soil total N and soil-available P were significant. It was also found that the mean observed overlaps of AMF phylotypes between the paired host plants in the same soil cores (27.1% of phylotypes shared) were significantly higher than the mean 1,000 simulated overlaps (14.2%). Furthermore, the same AMF sequences (100% sequence identity) were detected from both host plants in 8/12 soil cores having both roots. Accordingly, we concluded that *Py. pyrifolia* and *Pl. asiatica* examined shared some AMF communities, which suggested that understory herbaceous plants may function as AMF inoculum sources for orchard trees.

Arbuscular mycorrhizal fungi (AMF) are ubiquitous in most terrestrial plant communities and form mutualistic associations with the vast majority of plant species, including many cultivars (1, 36). This symbiosis is usually effective for growth promotion of host plants through improved mineral nutrients, particularly phosphorus (P).

It is well known that most orchard tree species have symbioses with AMF (4, 5, 12, 29, 30, 32). Orchard soil often contains an excess amount of fertilizer components, especially phosphorus, because of repeated fertilization (7, 25). Many studies have shown that the AMF colonization rate decreases with the addition of P to the soil (16, 17). In orchard environments, Youpensuk *et al.* (42) found that AMF root colonization rates and spore densities were significantly decreased in a tangerine (*Citrus reticulate*) orchard containing more than 500 mg P kg^−1^ of available P in soil. Rutto *et al.* (32) analyzed the effects of some cultural practices on AMF in various orchards and showed that excessive fertilization had adverse effects on AMF colonization and their spore populations. Thus, it is considered that AMF symbiosis is not fully utilized in plant cultivations in many orchards with excessive fertilization.

In orchards, various herbaceous plants are usually found around the trees, and many of them are known as AM plants (24). Sod culture is a cultivation method to manage orchards with herbaceous plants (35). Rutto *et al.* (32) found that the AMF colonization level and spore population in peach (*Prunus persica* Batsch) trees in sod culture were higher than those without cover plants, which suggested that sod culture may be effective to increase AMF colonization in orchard trees.

In this study, we examined AMF community in an orchard tree and a surrounding herbaceous plant. As the orchard tree species examined, we selected *Pyrus pyrifolia* var. *culta* (Rosaceae), known as Japanese pear. This is a well-known fruit tree species cultivated in various regions of Japan. In our previous study, it was found that AMF diversity and colonization were reduced by the accumulation of soil phosphorus in this orchard tree species (41). As the herbaceous plant species, we selected *Plantago asiatica* (Plantaginaceae), which is an herbaceous perennial that is often found in the understories of orchards. The Plantaginaceae are also known as hosts of AMF (22, 28).

In our previous observation, it was found that fine roots of *Py. pyrifolia* were sparsely distributed in soil, and new fine roots often arose from lignified small roots with no AMF colonization; therefore, it is considered that AMF colonizing roots of herbaceous plants such as *Pl. asiatica* could be important inocula for orchard trees such as *Py. pyrifolia*. Many studies have shown that host plant species are determinants of AMF communities (38, 39), Meanwhile, it has also been known that host specificity in AMF symbiosis is usually low (36). According to these studies, we developed a hypothesis that some AMF communities can be shared between the two host plants, *Py. pyrifolia* and *Pl. asiatica*, while they are diversified among the hosts. In order to test the hypothesis, we investigated AMF communities in the roots of both plants, *Py. pyrifolia* and *Pl. asiatica*, collected from the same soil cores to examine the overlap of AMF between the two host plants as well as to compare the AMF communities.

## Materials and Methods

### Sampling

Three orchards of Japanese pear, Nakayama (Nk; 35°30.7′ N, 133°33.9′ E), Yura (Yu; 35°28.5′ N, 133°44.5′ E), and Togo (Tg; 35°28.0′ N, 133°55.1′ E), in Tottori Prefecture were selected as the study sites, where sampling was conducted during June and July 2011. The soil type of the examined area was basically andosol caused by volcanic ash; however, the soil color was not blackish in most examined orchards because of the disappearance of topsoil during the preparation of orchards. Five trees of *Py. pyrifolia* var. *culta* cultivar ‘Osa-Gold’ were randomly selected in each orchard, and three individuals of *Pl. asiatica*, located approximately 100 cm from the tree base, were selected for each tree. There were many species of herbaceous plants in the orchards, in which *Pl. asiatica* was found in all the examined orchards. A soil core sample (5 cm in diameter and 10 cm in depth) was collected under the shoot of each selected *Pl. asiatica* plant; thus, 45 soil cores were selected in total. Ten leaves of *Py. pyrifolia* were also collected from the middle of the spurs on each tree selected.

### AMF colonization rate

The roots of *Py. pyrifolia* (thin, yellowish or brownish, and less branched) and *Pl. asiatica* (white and connected to the shoot) were isolated from the soil core samples, and washed carefully with tap water to remove the attached soil, and then the fine roots were collected. Around 5–30 mg of the fresh fine roots of *Py. pyrifolia* and around 20–500 mg of *Pl. asiatica* were used to determine the AMF colonization rates according to the method of Brundrett *et al.* (2) with slight modifications as follows. The fine roots were cleared in 10% KOH at 121°C for 20 min by autoclaving. After sequential rinsing with distilled water, alkaline H_2_O_2_, and 2% HCl, the roots were stained with 0.05% trypan blue at 100°C in a water bath for 10 min. The stained roots were then stored in a lactic acid:glycerol:water (1:1:1) solution. The AMF colonization rate was determined by the gridline intersection method with at least 100 intersections.

### Soil chemical analysis

The soil samples collected from the soil cores were analyzed to determine soil chemical properties. The soil pH (H_2_O) at 1:2.5 soil:water ratio and the available P (0.002N H_2_SO_4_ extractable P, Truog method) were measured. Total C and total N were analyzed using an Elementar Vario EL CHNS analyzer (Elementar, Hanau, Germany).

### Leaf P content

The leaves collected from both host plants were washed with tap water, rinsed with distilled water, and dried at 70°C for 48 h. The leaves were then ground using an Oster Blender (Osaka Chemical, Osaka, Japan) and digested using H_2_SO_4_ and H_2_O_2_ in a heat block at approximately 200°C. Subsequently, the leaf P content was determined using the vanadomolybdate spectrophotometric method (26).

### Molecular analysis of AMF

Fresh fine roots, around 10–100 mg from *Py. pyrifolia* and around 50–800 mg from *Pl. asiatica* were used to analyze the AMF communities. DNA was extracted using a DNeasy Plant Mini Kit (Qiagen, Tokyo, Japan). The AMF communities were examined on the basis of the partial fungal DNA sequences (approximately 750 bp) of the nuclear small subunit ribosomal RNA gene (SSU rDNA), which was amplified by nested polymerase chain reaction (PCR) as follows. The universal primer set NS1 (13) and NS4 (40) was used in the first PCR. The reaction mixture contained 1.0 μL of extracted DNA, 0.15 μL of Taq DNA polymerase, 3 μL of PCR buffer (100 mM Tris-HCl [pH 8.3], 500 mM KCl, and 15 mM MgCl_2_), 2.4 μL of each deoxynucleotide triphosphate, and 0.15 μM of each primer in a total volume of 30 μL. The PCR program was as follows: an initial denaturation step at 94°C for 2 min, followed by 30 cycles of 94°C for 30 s, 40°C for 1 min, and 72°C for 1 min, and a final elongation step at 72°C for 10 min (PC-818S Program Temp Control System; Astec, Fukuoka, Japan). In the second PCR, 1 μL of a 1:10 dilution of the first PCR product was used with the AMF-specific primers AML1 and AML2 (21) in the same reaction mixture. The PCR program was as follows: an initial denaturation step at 94°C for 2 min, followed by 35 cycles at 94°C for 30 s, 50°C for 30 s, and 72°C for 1 min, and a final elongation step at 72°C for 10 min. The PCR products were purified using a Gel/PCR DNA Fragment Extraction Kit (RBC Bioscience, Taipei, Taiwan) and cloned using a pGEM-T Easy Vector System I (Promega, Madison, WI, USA), according to the manufacturer’s instructions. The clones were analyzed by colony PCR, which was followed by RFLP using *Hinf*I to exclude plant sequences from further analysis. The DNA inserts were sequenced using the BigDye Terminator v3.1 Cycle Sequencing Kit (Applied Biosystems, Foster City, CA, USA) with the promoter primer T7 on a 3130 Genetic Analyzer (Hitachi, Tokyo, Japan). Multiple sequence alignments were performed using ClustalX version 2.0.12 for all sequenced data (20). The aligned sequences were analyzed using the neighbor-joining method (33) with 1,000 bootstrap analysis (10), and the phylogenetic trees were drawn using TreeView (27). The AMF phylotypes were defined on the basis of tree topology and sequence similarity computed using ClustalX. A rarefaction curve was computed for each sample by plotting the number of AMF phylotypes detected against the number of sequences using Analytic Rarefaction version 1.3 (https://www.uga.edu/_strata/software/AnRare/Readme.html). After selecting representative sequences of each phylotype, BLAST searches were performed to download similar sequences. Multiple sequence alignments, neighbor-joining analysis, and phylogenetic tree drawing were performed as described above for the sequenced and downloaded data. Furthermore, the pairwise sequence similarities between AMF from *Py. Pyrifolia* and *Pl. asiatica* in the same soil cores were computed using ClustalX.

### Data analysis

The relationships between the soil-available P or leaf P and AMF colonization rates or the number of AMF phylotypes were investigated by Pearson’s correlation coefficient test.

The relationships between the AMF community and environmental variables were tested using multivariate analyses with CANOCO 4.5 (37). In the data table of the response variables, *i.e.*, the distribution of AMF phylotypes, the presence or absence of each phylotype in each sample was scored with the dummy variables “1” or “0.” The AMF phylotypes detected in at least 3 samples were adopted for the analysis. In the explanatory (environmental) variable data, the soil chemical properties (pH, available P, total C, and total N) were used as quantitative variables. The host plant species (*Py. pyrifolia* and *Pl. asiatica*) and the examined orchards (Nakayama, Yura, and Togo) were used as nominal variables.

Detrended correspondence analysis (DCA) was applied to the response variable data in order to estimate the heterogeneity based on the length of the community composition gradients in species turnover units. DCA was performed with detrending by segments. After confirming the length of the composition gradients on the first DCA axis, correspondence analysis (CA) was performed to infer the relationships between AMF distribution and environmental variables. CA was performed with scaling to interspecies correlations with division by standard deviation and centering by species. The resulting diagram for samples was visualized using CanoDraw.

To explain the effects of environmental variables, canonical correspondence analysis (CCA) was performed with scaling to interspecies correlations with division by standard deviation and centering by species. Monte Carlo permutation tests with 999 unrestricted permutations were then performed for manually selected environmental variables to evaluate their effects on the AMF community.

In order to examine whether the overlap of AMF phylotypes on the two host plants *Py. pyrifolia* and *Pl. asiatica* was significantly different from what would be expected by random chance, the overlap in the soil cores was analyzed by Ecosim Professional (9). The roots of both plants collected from the same soil cores were paired, and the overlap of AMF phylotypes in the pairs was examined by the species overlap module in the program. In this analysis, the presence or absence of each phylotype was scored with “1” or “0”, and the observed mean overlap between the two host plants was compared to the 1,000 simulated mean overlap.

## Results

### Molecular analysis of the AMF community

**Fig. 1:**
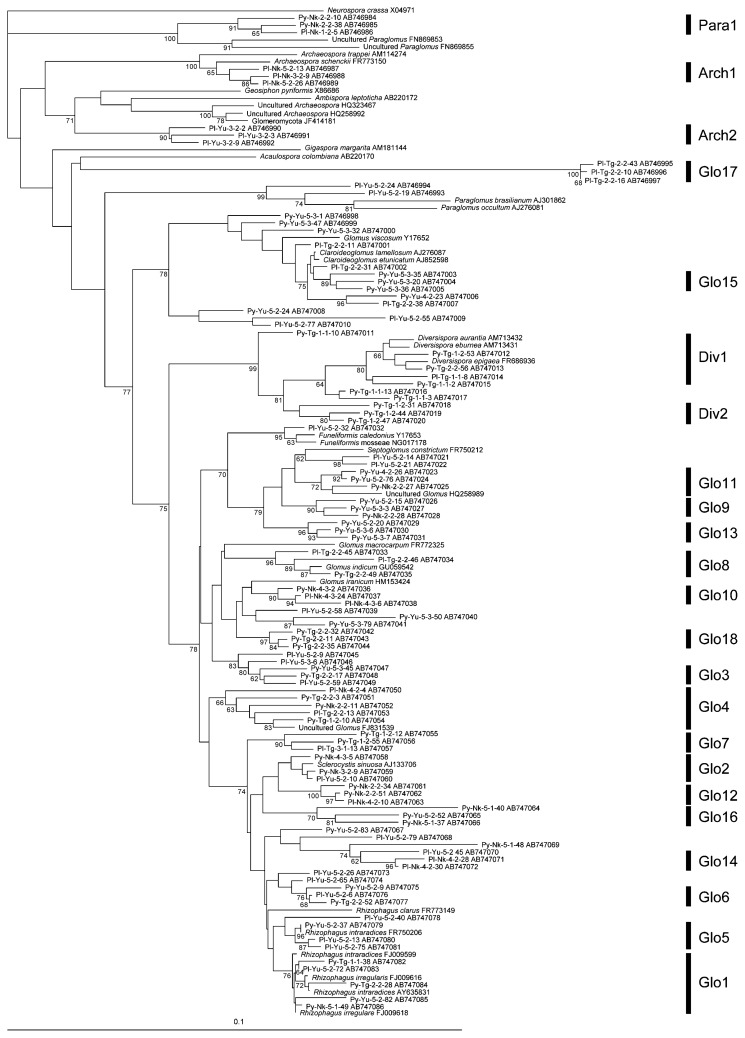
Neighbor-joining phylogenetic tree based on the partial sequences of SSU rDNA from arbuscular mycorrhizal fungi (AMF) in the roots of *Pyrus pyrifolia* var. *culta* (Japanese pear) and *Plantago asiatica*, and from the GenBank database. The tree is rooted by *Neurospora crassa* (X04971) in the *Ascomycota*. Sequence numbers refer to the host plant, orchard, tree, soil core, and clone numbers. The division of phylotypes (Glo1–Glo18, Arch1–Arch2, Div1–Div2, and Para1) is shown. Bootstrap values exceeding 70% are shown (1,000 replicates). The scale is shown so that the evolutionary distances can be inferred. Accession numbers are given for all sequences.

Sufficient fine roots for the examination were obtained from 16 samples of *Py. pyrifolia* (8 samples from Nakayama, 4 from Yura, and 4 from Togo) and 13 samples of *Pl. asiatica* (7 samples from Nakayama, 3 from Yura, and 3 from Togo) soil cores. Accordingly, molecular analysis of the AMF communities was performed on these 29 root samples. After cloning the PCR products, 14–79 clones were sequenced for each sample to obtain 811 (497 from *Py. pyrifolia* and 314 from *Pl. asiatica*) sequences in total. Neighbor-joining phylogenetic analysis was performed for all the AMF sequences obtained, and the phylotypes were determined to have a sequence similarity of >96% with at least three sequences. Consequently, 777 sequences were divided into 23 phylotypes. A neighbor-joining tree for the representative and downloaded AMF sequences is shown in Fig. 1. For the taxonomy of the identified AMF whose sequences were downloaded from GenBank, new revised genera based on the molecular phylogeny (19, 34) were shown in the phylogenetic tree; however, allocation to the new genera was difficult for most of the phylotypes in this study because of the lack of known species phylogenetically related. Thus, we used a former taxonomy based on the spore morphology to allocate the phylotypes. Consequently, 18 phylotypes were identified as *Glomus*, two each were *Archaeospora* and *Diversispora*, and one was *Paraglomus*. The phylogenetic tree showed that about half (12/23) of the phylotypes included AMF from both host plants, *Py. pyrifolia* and *Pl. asiatica*. Of the 12 phylotypes, 85.0% (689/811) of the AMF sequences obtained were affiliated (Fig. 1).

### AMF colonization rates, number of phylotypes, soil chemical properties, and leaf P

The AMF colonization rates, number of AMF phylotypes, soil chemical properties, and leaf P are shown in Table 1. The AMF colonization rate was determined for the 29 root samples described above. The rates for *Py. pyrifolia* and *Pl. asiatica* were 21.7–79.2% and 0–67.8%, respectively. The soil-available P were 100.5–1,324 mg P kg^−1^. The leaf P was 2.9–4.1 mg P g^−1^ for *Py. pyrifolia* and 6.9–25.0 mg P g^−1^ for *Pl. asiatica*. Among the correlations between soil P or leaf P and AMF colonization rates or the number of AMF phylotypes, significant correlation was only found between the leaf P and the number of AMF phylotypes in *Pl. asiatica* (R=0.62, *P*<0.05). A significant correlation was also found between the number of phylotypes and the AMF colonization rate in *Py. pyrifolia* (R=0.66, *P*<0.01).

### AMF community and environmental variables

**Fig. 2:**
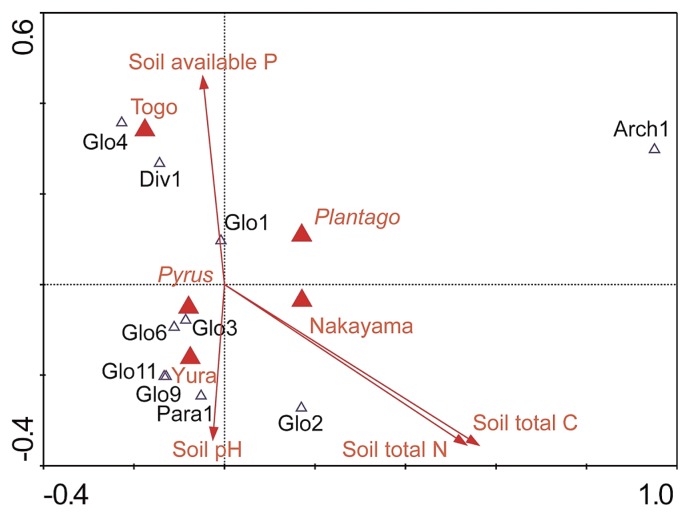
Diagram of correspondence analysis (CA) of the communities of arbuscular mycorrhizal fungi (AMF) found in the two host plants, *Pyrus pyrifolia* var. *culta* and *Plantago asiatica* in three examined orchards (Nakayama, Yura, and Togo). As environmental variables, soil chemical properties, soil-available P, soil pH, soil total N, and soil total C were examined as well as the host plants and the examined orchards. The eigenvalues of the first and second PCA axes were 0.695 and 0.568, respectively.

DCA of the response variable data showed that the length of the community composition gradient of the first axis was 4.68. Based on this result, we used CA as the unimodal ordination method. The resulting ordination is shown in Fig. 2. The eigenvalues of the first and second axes were 0.695 and 0.568, respectively. The cumulative percentage variance of the species data showed that the first two CA axes explained 41.5% of the variability in the species data. The ordination diagram (Fig. 2) suggested that most AMF preferred a lower soil-available P condition. The Monte Carlo permutation tests on CCA showed that the three examined orchards (Nakayama, Yura, and Togo) and the soil chemical properties (total C, total N, and available P) had significant relationships with the distribution of AMF phylotypes (*P*<0.05) (Table 2). In contrast, the effects of the two host plant species (*Py. pyrifolia* and *Pl. asiatica*) were not significant (Table 2).

### AMF overlap between paired host plants

Twelve *Py. pyriforia-Pl. asiatica* pairs were made from 12 soil cores, and the species overlap module was performed on the AMF phylotype data matrix. The mean observed overlap of AMF phylotypes (27.1% of species shared) was significantly higher than the mean 1,000 simulated overlap (14.2%) (*P*=0.002).

### Co-occurrence of AMF in both host plants

AMF were detected from both *Py. pyrifolia* and *Pl. asiatica* in 12 soil cores, and the same AMF sequences (100% identity) were detected in 8 of them. The proportion of AMF sequences in *Py. pyrifolia* that shared 100% identity with AMF sequences in *Pl. asiatica* in these 8 soil cores ranged from 2.1 to 85.7% with an average of 22.1% (Table S1). Similarly, the proportion of AMF sequences in *Pl. asiatica* that shared 100% identity with those in *Py. pyrifolia* ranged from 3.6 to 92.9% with an average of 35.4% (Table S1).

## Discussion

AMF colonization and communities in roots of *Py. pyrifolia* and *Pl. asiatica* collected from the same soil cores were investigated in this study. Among the correlation analyses, a positive significant correlation was found between leaf P and the number of AMF phylotypes in *Pl. asiatica*. Because higher P in plants usually has negative effects on AMF symbioses, further investigation may be required for this phenomenon. The number of phylotypes and the AMF colonization rate in *Py. pyrifolia* were also significant. This may indicate the importance of AMF diversity for symbiosis.

Most AMF detected in the roots of the two host plants in this study were *Glomus* spp., whereas *Paraglomus*, *Archaerospora*, and *Diversispora* spp. were also detected (Fig. 1). The PCR primer set AML1 and AML2 can amplify the sequences of most AMF (21), and their adaptation for diverse AMF taxa was confirmed in this study; therefore, the dominant detection of *Glomus* fungi in the roots of *Py. pyrifolia* and *Pl. asiatica* can be a reflection of the actual AMF communities.

Phylogenetic analysis showed that AMF from both host plants were divided into 23 phylotypes (Fig. 1). Among them, 7 phylotypes were detected only from *Py. pyrifolia*, while 4 were only from *Pl. asiatica*, which suggested that the increased plant species richness could increase AMF diversity. Similar effects of increased plant species on AMF diversity have been shown in some other studies (3, 6). Meanwhile, 12 phylotypes included AMF sequences from both host plants, and 689/811 AMF sequences were affiliated to these 12 phylotypes. This result indicated that dominant AMF could colonize both plants.

The CCA also showed that host plant species did not have significant effects on the distribution of AMF phylotypes, whereas the effects of sampling site, soil total C, soil total N, and soil-available P were significant (Table 2). This result suggested that AMF communities are less diversified between the two host plants.

To examine the hypothesis that some AMF can be shared between the host plants, AMF overlap between two hosts in each soil core was examined. The result showed that the observed overlap (27.1% of AMF phylotypes shared) was significantly higher than what would be expected by random chance (14.2%). Furthermore, the same AMF sequences with 100% sequence identity were found in both plants in 8/12 soil cores (Table S1). Thus, it was concluded that *Py. pyrifolia* and *Pl. asiatica* in the orchards shared some AMF communities.

AMF sharing among different plant species may have some physiological or ecological significance (14). The AMF mycelium network can promote the colonization of other plants growing around them (11, 23, 31). Enkhtuya and Vosátka (8) showed that AMF colonization extends from the herbaceous plant *Agrostis capillaries* to tree seedlings by an extraradical mycelium, in which P transport in the hyphal network was confirmed. Trees also could act as reservoirs of AMF for surrounding herbaceous plants (15). These results suggested that surrounding herbaceous plants and trees can act as mutual reservoirs of AMF to maintain the mycelium network. The genus *Pyrus* extends the fine roots (<1 mm) sparsely (18) and their new fine roots often arise from lignified small roots with no AMF colonization; thus, it is supposed that the AMF colonizing the surrounding herbaceous plants could serve as inocula for the roots of *Py. pyrifolia*. The significant overlap of AMF in the same cores may suggest this interaction.

We studied AMF of *Pl. asiatica*, one of the herbaceous perennials found around pear trees, because this plant species is common in orchards. Indeed, *Pl. asiatica* was found in all three of the orchards examined in this study; however, the actual herbaceous plant communities found in orchards contain diverse plant species, which could vary depending on the environmental conditions and agricultural practices. In order to consider the utilization of herbaceous plants as inocula of AMF for orchard trees, further study is required for the AMF in herbaceous plant communities.

## Supplementary Material



## Figures and Tables

**Table 1:** Soil chemical properties, leaf P, AMF colonization rate, and number of phylotypes of *Pyrus pyrifolia* and *Plantago asiatica* in each sample

Site	Soil core sample*	Soil				*Py. pyrifolia*			*Pl. asiatica*		
		
		pH	Available P (mg P kg^−1^)	Total C (g kg^−1^)	Total N (g kg^−1^)	Leaf P (mg P g^−1^)	AMF colonization rate (%)	Number of AMF phylotypes	Leaf P (mg P g^−1^)	AMF colonization rate (%)	Number of AMF phylotypes
Nakayama	Nk-1-2	6.1	390.8	66.9	4.87	3.8	26.0	2	11.0	16.2	2
	Nk-2-2	6.4	528.6	62.0	4.80	2.9	49.9	5	—	—	—
	Nk-3-2	6.2	480.1	86.2	6.43	3.4	30.7	2	9.3	13.3	1
	Nk-4-2	6.2	284.1	51.0	4.10	4.1	22.3	1	25.0	45.2	4
	Nk-4-3	5.9	761.5	51.4	4.50	4.1	42.8	3	14.1	67.8	1
	Nk-5-1	5.9	586.8	92.6	6.93	3.8	52.3	3	6.9	33.2	1
	Nk-5-2	5.7	747.9	92.1	6.47	3.8	35.4	1	11.3	59.1	2
	Nk-5-3	6.3	563.5	85.3	6.30	3.8	24.8	1	12.3	45.1	2
Yura	Yu-3-2	5.8	998.2	43.9	4.43	—**	—	—	7.2	51.9	2
	Yu-4-2	6.2	998.2	67.6	5.47	3.0	61.5	3	—	—	—
	Yu-4-3	5.5	714.9	36.9	3.63	3.0	21.7	1	—	—	—
	Yu-5-2	6.3	1324	62.8	4.90	3.2	79.2	10	14.2	66.0	5
	Yu-5-3	6.1	901.2	33.6	3.03	3.2	66.8	8	8.1	54.1	2
Togo	Tg-1-1	5.9	348.9	28.1	2.60	3.7	45.0	2	7.2	41.0	1
	Tg-1-2	5.6	148.4	26.9	2.63	3.7	68.7	5	—	—	—
	Tg-2-2	6.0	382.5	57.3	4.87	3.7	30.2	7	11.2	49.5	3
	Tg-3-1	5.5	100.5	17.5	2.10	3.0	57.1	1	7.7	16.6	2

* The sample name refers to the orchard, tree number, and soil core number.

** Samples with fewer fine roots were not analyzed.

**Table 2:** Results of Monte Carlo permutation tests (999 permutations) on redundancy analysis (CCA) for the effect of sites, soil chemical properties, and host plants on arbuscular mycorrhizal fungal community

Environmental variables	*F* value	*P* value
Nakayama	2.82	0.004
Togo	3.11	0.002
Yura	2.89	0.004
Soil total C	3.07	0.001
Soil total N	2.87	0.003
Soil-available P	1.84	0.048
Soil pH	1.42	0.161
*Plantago*	1.69	0.073
*Pyrus*	1.69	0.076
