# Supplementary material for: Communities of Arbuscular Mycorrhizal Fungi in Pyrus pyrifolia var. culta (Japanese pear) and an Understory Herbaceous Plant Plantago asiatica
Source: Microbes Environ. 2013 Apr 24;28(2):204–10. doi: 10.1264/jsme2.ME12180 (PMC4070674; doi:10.1264/jsme2.ME12180)
Supplement: Supplementary file 1 [file 28_204_s1.pdf]

Table S1 AMF phylotypes detected in roots of *Pyrus pyrifoloa* var. *culta* and *Plantago asiatica* and their co-occurrence in both plants in 12 soil core samples

Soil core	AMF phylotypes (*Py. pyrifolia*)	AMF phylotypes (*Pl. asiatica*)
Nk-1-2	Glo1 Glo1 Glo1 Glo1 Glo1 Glo1 Glo1 Glo1 Glo1 Glo1<br>Glo1 Glo1 Div1 Div1 Div1 Div1 Div1 Div1<br>50% (9/18)*	Glo1 Glo1 Glo1 Glo1 Glo1 Glo1 Glo1 Glo1 Glo1 Glo1<br>Glo1 Glo1 Para1 Para1 Para1<br>66.7% (10/15)
Nk-3-2	Glo2 Glo2 Glo2 Glo2 Glo2 Glo2 Glo2 Glo2 Glo2 Glo2<br>Glo2 Glo2 Glo2 Glo2 Para1<br>0% (0/15)	Arch1 Arch1 Arch1 Arch1 Arch1 Arch1 Arch1 Arch1 Arch1 Arch1<br>Arch1 Arch1 Arch1 Arch1 Arch1 Arch1<br>0% (0/16)
Nk-4-2	Glo1 Glo1 Glo1 Glo1 Glo1 Glo1 Glo1 Glo1 Glo1 Glo1<br>Glo1 Glo1 Glo1 Glo1<br>85.7% (12/14)	Glo1 Glo1 Glo1 Glo1 Glo1 Glo1 Glo1 Glo1 Glo1 Glo1<br>Glo1 Glo1 Glo1 Glo1 Glo1 Glo1 Glo1 Glo1 Glo1 Glo1<br>Glo1 Glo1 Glo1 Glo1 Glo1 Glo1 Glo4 Glo4 Glo4 Glo12<br>Glo14 Glo14 Glo14<br>60.6% (20/33)
Nk-4-3	Glo1 Glo1 Glo2 Glo2 Glo2 Glo2 Glo2 Glo2 Glo2 Glo2<br>Glo2 Glo2 Glo2 Glo2 Glo10<br>0% (0/15)	Glo10 Glo10 Glo10 Glo10 Glo10 Glo10 Glo10 Glo10 Glo10 Glo10<br>Glo10 Glo10 Glo10 Glo10 Glo10 Glo10 Glo10<br>0% (0/17)
Nk-5-1	Glo1 Glo1 Glo1 Glo1 Glo1 Glo1 Glo1 Glo1 Glo1 Glo1<br>Glo1 Glo1 Glo1 Glo1 Glo1 Glo1 Glo1 Glo2 Glo2 Glo2<br>Glo2 Glo2 Glo2 Glo2 Glo2 Glo2 Glo2 Glo2 Glo2 Glo2<br>Glo2 Glo2 Glo16 Glo16<br>20.6% (7/34)	Glo2 Glo2 Glo2 Glo2 Glo2 Glo2 Glo2 Glo2 Glo2 Glo2<br>Glo2 Glo2 Glo2 Glo2<br>92.9% (13/14)
Nk-5-2	Glo2 Glo2 Glo2 Glo2 Glo2 Glo2 Glo2 Glo2 Glo2 Glo2<br>Glo2 Glo2 Glo2 Glo2 Glo2 Glo2 Glo2 Glo2<br>55.6% (10/18)	Glo2 Glo2 Glo2 Glo2 Glo2 Glo2 Glo2 Glo2 Arch1 Arch1<br>Arch1 Arch1 Arch1 Arch1 Arch1<br>26.7% (4/15)
Nk-5-3	Glo2 Glo2 Glo2 Glo2 Glo2 Glo2 Glo2 Glo2 Glo2 Glo2<br>Glo2 Glo2 Glo2 Glo2 Glo2 Glo2 Glo2 Glo2 Glo2<br>0% (0/19)	Glo1 Glo1 Glo1 Glo1 Glo1 Glo1 Glo1 Glo1 Glo1 Glo1<br>Glo1 Glo1 Glo1 Glo1 Glo1 Glo1 Glo1 Glo1 Glo1 Glo1<br>Glo1 Arch1 Ot1**<br>0% (0/23)
Tg-1-1	Glo1 Glo1 Glo1 Glo1 Glo1 Glo1 Glo1 Glo1 Glo1 Glo1<br>Glo1 Glo1 Glo1 Glo1 Glo1 Glo1 Glo1 Glo1 Glo1 Div1<br>Div1 Div1 Div1 Div1 Div1 Div1 Div1 Div1 Div1 Div1<br>Div1 Div1 Div1 Ot1 Ot2<br>8.6% (3/35)	Div1 Div1 Div1 Div1 Div1 Div1 Div1 Div1 Div1 Div1<br>Div1 Div1 Div1 Div1<br>92.9% (13/14)
Tg-2-2	Glo1 Glo3 Glo3 Glo4 Glo4 Glo4 Glo4 Glo4 Glo6 Glo6<br>Glo6 Glo6 Glo6 Glo8 Glo8 Glo8 Glo8 Glo8 Glo8 Glo8<br>Glo6 Glo6 Glo8 Glo8 Glo8 Glo8 Glo8 Glo8 Glo8 Glo8<br>Glo8 Glo8 Glo8 Glo8 Glo8 Glo8 Glo8 Glo8 Glo8 Glo8<br>Glo18 Glo18 Glo18 Div1 Ot1 Ot2 Ot3 Ot4<br>2.1% (1/48)	Glo4 Glo4 Glo4 Glo4 Glo4 Glo4 Glo4 Glo4 Glo4 Glo4<br>Glo8 Glo8 Glo8 Glo8 Glo8 Glo8 Glo8 Glo8 Glo8 Glo8<br>Glo8 Glo8 Glo17 Glo17 Glo17 Ot1 Ot2 Ot3<br>3.6% (1/28)
Tg-3-1	Glo4 Glo4 Glo4 Glo4 Glo4 Glo4 Glo4 Glo4 Glo4 Glo4<br>Glo4 Glo4 Glo4 Glo4 Glo4 Glo4 Glo4 Glo4 Glo4 Glo4<br>Glo4 Glo4 Glo4 Glo4 Glo4 Glo4 Glo4 Glo4<br>0% (0/28)	Glo1 Glo7 Glo7 Glo7 Glo7 Glo7 Glo7 Glo7 Glo7 Glo7<br>Glo7 Glo7 Glo7 Glo7 Glo7 Glo7 Glo7 Glo7 Glo7 Glo7<br>Glo7 Glo7 Glo7 Glo7 Glo7 Ot1<br>0% (0/26)
Yu-5-2	Glo1 Glo1 Glo1 Glo1 Glo1 Glo1 Glo1 Glo1 Glo1 Glo1<br>Glo2 Glo2 Glo2 Glo2 Glo2 Glo2 Glo2 Glo2 Glo2 Glo2<br>Glo2 Glo2 Glo2 Glo2 Glo2 Glo2 Glo2 Glo2 Glo3 Glo3<br>Glo3 Glo3 Glo3 Glo3 Glo3 Glo3 Glo3 Glo3 Glo5 Glo5<br>Glo5 Glo5 Glo5 Glo5 Glo5 Glo5 Glo5 Glo5 Glo5 Glo5<br>Glo5 Glo5 Glo5 Glo5 Glo6 Glo6 Glo6 Glo6 Glo6 Glo6<br>Glo6 Glo6 Glo6 Glo6 Glo7 Glo9 Glo9 Glo9 Glo9 Glo11<br>Glo11 Glo11 Glo13 Glo16 Ot1<br>36% (27/75)	Glo1 Glo1 Glo2 Glo2 Glo2 Glo2 Glo2 Glo2 Glo2 Glo2<br>Glo2 Glo2 Glo3 Glo3 Glo3 Glo5 Glo5 Glo5 Glo5 Glo5<br>Glo5 Glo5 Glo5 Glo5 Glo5 Glo5 Glo5 Glo5 Glo5 Glo5<br>Glo5 Glo5 Glo5 Glo5 Glo5 Glo5 Glo5 Glo5 Glo5 Glo5<br>Glo5 Glo5 Glo5 Glo5 Glo5 Glo5 Glo5 Glo5 Glo5 Glo5<br>Glo5 Glo5 Glo5 Glo6 Glo6 Glo6 Glo6 Glo6 Glo6 Glo6<br>Glo6 Glo6 Glo6 Glo6 Glo6 Glo6 Glo6 Ot1 Ot2 Ot3<br>Ot4 Ot5 Ot6 Ot7 Ot8 Ot9 Ot10 Ot11 Ot12<br>35% (28/79)
Yu-5-3	Glo1 Glo1 Glo1 Glo1 Glo2 Glo2 Glo2 Glo2 Glo2 Glo2<br>Glo2 Glo2 Glo2 Glo2 Glo2 Glo2 Glo2 Glo2 Glo2 Glo2<br>Glo3 Glo3 Glo3 Glo3 Glo3 Glo3 Glo3 Glo3 Glo3 Glo3<br>Glo3 Glo3 Glo3 Glo3 Glo3 Glo3 Glo3 Glo3 Glo3 Glo3<br>Glo3 Glo3 Glo3 Glo3 Glo3 Glo3 Glo3 Glo3 Glo3 Glo3<br>Glo3 Glo3 Glo9 Glo9 Glo9 Glo9 Glo9 Glo11 Glo11 Glo13<br>Glo13 Glo15 Glo15 Glo15 Para1 Ot1 Ot2 Ot3 Ot4 Ot5<br>Ot6 Ot7<br>5.7% (4/72)	Glo1 Glo1 Glo1 Glo1 Glo1 Glo1 Glo1 Glo1 Glo1 Glo3<br>Glo3 Glo3 Glo3 Glo3<br>50% (7/14)

The phylotypes having 100 sequence identities with those of another plant in the same soil core were shown with gray.

*The ratio of AM fungal sequences having 100% identity with those of another plant.

**Ot: Other sequences that were not included into any phylotypes.
